# IMP4 Silencing Inhibits the Malignancy of Lung Adenocarcinoma via ERK Pathway

**DOI:** 10.1155/2022/8545441

**Published:** 2022-10-22

**Authors:** Ruzhen Li, Zhaohui Han, Wei Ma, Lin Zhang, Xiangwei Zhang, Yuanzhu Jiang, Wei Dong

**Affiliations:** Department of Thoracic Surgery, Shandong Provincial Hospital Affiliated to Shandong First Medical University, Jinan, China

## Abstract

Our study aimed to elucidate the function of IMP U3 small nucleolar ribonucleoprotein 4 (IMP4) in lung adenocarcinoma (LUAD) and its potential molecular mechanisms. Cell counting kit-8, 5-ethynyl-20-deoxyuridine, flow cytometry, wound healing, and transwell assays were performed to examine the biological behaviour of LUAD cells. mRNA and protein expression levels were determined using quantitative real-time PCR, Western blotting, and immunohistochemistry. In addition, a mouse tumour xenograft model was used to evaluate the role of IMP4 in tumour progression. Furthermore, glycolysis-related indicators were measured. The levels of IMP4 were up-regulated in both human LUAD tissues and cells. IMP4 silencing significantly suppressed proliferation, migration, invasion, and glycolysis; promoted apoptosis; and induced cell cycle arrest in LUAD cells. IMP4 silencing also inactivated the extracellular signal-regulated kinase (ERK) pathway. Moreover, rescue experiments demonstrated that the function of LUAD cells induced by IMP4 overexpression could be reversed by treatment with an ERK pathway inhibitor (SCH772984). *In vivo* experiments further verified that IMP4 silencing repressed the growth of subcutaneous tumours and glycolysis. IMP4 silencing suppressed the malignancy of LUAD by inactivating ERK signalling.

## 1. Introduction

Lung adenocarcinoma (LUAD) is a histological phenotype of lung cancer and one of the leading causes of cancer-related mortality and morbidity worldwide [1, 2]. Despite the progress in prevention, detection, and treatment of LUAD, the prognosis of LUAD patients remains poor. Therefore, there is an urgent need to explore the potential molecular mechanisms of LUAD and identify new therapeutic targets for LUAD.

IMP U3 small nucleolar ribonucleoprotein 4 (IMP4) is a component of U3 small nucleolar ribonucleoproteins and is involved in the maturation of 18S rRNA [3]. Moreover, it has been reported that IMP4 is a new telomeric DNA-binding protein, which can play important roles in telomeres [3]. Recently, a study by Chang et al. showed that IMP4, as a potential passenger gene, may be involved in endometrial cancer [4]. Moreover, IMP4 was shown to be up-regulated and was verified to be a novel target for lung cancer therapy [5]. However, the specific effects and molecular mechanisms of IMP4 in LUAD have not been fully explored.

Aerobic glycolysis is a hallmark of cancer [6, 7]. Accumulating studies show that aerobic glycolysis plays a critical role in uncontrolled proliferation, metabolism, and metastasis of tumour cells [8]. Interestingly, extracellular signal-regulated kinase (ERK) signalling is required for the responses to extracellular stimuli and has recently emerged as an important modulator of glycolysis during tumorigenesis [9]. Du et al. [10] reported that ATPR can induce acute myeloid leukaemia cell differentiation and cycle arrest by regulating the ERK-glycolysis signalling axis. However, it is unclear whether IMP4 plays a role in LUAD by regulating glycolysis and ERK signalling.

In this study, we showed that IMP4 could promote the progression of LUAD through activating the ERK pathway. These findings demonstrate the functions of IMP4 in LUAD and highlight its potential as a biomarker for LUAD.

## 2. Materials and Methods

### 2.1. Cell Culture and Transfection

Human LUAD cell lines (NCI–H1975, H1299, A549, and H23) and the human bronchial epithelioid cell line 16-HBE were purchased from Procell (Wuhan, China) and maintained in a RPMI-1640 medium (Hyclone) supplemented with 10% fetal bovine serum (FBS; FS301-02, Transgen, Beijing, China) and 1% streptomycin/penicillin (P1400, Solarbio, Beijing, China) in a humidified atmosphere of 5% CO_2_ at 37°C. IMP4 siRNA-1, IMP4 siRNA-2, and IMP4 siRNA negative controls were obtained from Ribobio (Guangzhou, China), while pcDNA3.1-IMP4, pcDNA3.1-E2F4, and its control vector were supplied by Tsingke Biotechnology Co., Ltd. (Beijing, China). Based on the manufacturer's instructions, IMP4 siRNA-1 (si-IMP4-1), IMP4 siRNA-2 (si-IMP4-2), IMP4 siRNA negative control (si-NC), pcDNA3.1-IMP4 (IMP4), pcDNA3.1-E2F4 (E2F4), and empty pcDNA3.1 vector (Vector) were transfected into cells for two days.

### 2.2. Cell Counting Kit-8 (CCK-8) Assay

The viability of A549 and H1299 cells was evaluated using the CCK-8 kit (C0037, Beyotime, Shanghai, China) according to the manufacturer's instructions, as described previously [11].

### 2.3. 5-ethynyl-20-deoxyuridine (EdU) Assay

Transfected A549 and H1299 cells (2 × 10^3^ cells/well) were seeded into 6-well plates and subsequently incubated with EdU solution (C0071S, Beyotime, Shanghai, China) for 120 min. DAPI (C1002, Beyotime) was then applied to stain cell nuclei for 30 min in the dark. Finally, a fluorescence microscope was used to analyse the EdU-positive cells.

### 2.4. Flow Cytometry

After transfection for 48 h, the A549 and H1299 cells were harvested, washed twice with ice-cold PBS, and resuspended in 1× binding buffer. For cell apoptosis analysis, cells were incubated in Annexin-V/PI double staining solution (KGA101, KeyGen Biotech, Nanjing, China) for 20 min, according to the manufacturer's instructions, and then analysed using a FACSCalibur flow cytometer. The cell cycle was evaluated using a cell cycle and apoptosis analysis kit (C1052, Beyotime) according to the manufacturer's instructions using a FACS Calibur flow cytometer.

### 2.5. Wound Healing Assay

After transfection for 48 h, A549 and H1299 cells (4 × 10^5^ cells/well) were seeded in a 6-well plate. Once the A549 and H1299 cells had grown to 100% confluence, wounds in the monolayer were created using a sterile pipette tip, and then the wound's distance at 0 h and 24 h was photographed using a light microscope.

### 2.6. Transwell Assay

The migration and invasion abilities of A549 and H1299 cells were evaluated using a Transwell™ chamber (pore size, 8 *μ*m; CLS3422, Corning, USA). Briefly, 2 × 10^4^ A549 and H1299 cells were plated into the upper chamber, which was precoated with Matrigel (BD 354230, BD Biosciences, USA.). Subsequently, 800 *μ*L of the medium was added to the bottom chamber. After 24 h, the cells that migrated across the membrane were stained with crystal violet (C0121, Beyotime). Finally, the cell numbers in five random fields of view were observed using a microscope.

### 2.7. Glucose Uptake, Lactate, and Adenosine Triphosphate (ATP) Assays

The levels of glucose, lactate, and adenosine triphosphate (ATP) were measured using a glucose colorimetric assay kit (MAK083, Sigma-Aldrich, USA), a lactate assay kit (KGT02424, KeyGen Biotech), and an enhanced ATP assay kit (S0026, Beyotime) according to the manufacturer's instructions.

### 2.8. RNA Extraction and qRT-PCR

Total RNA was obtained using TRIZOL (R0016, Beyotime) and then reverse transcribed into complementary DNA using *EasyScript*® First-Strand cDNA Synthesis SuperMix (AE301-02, TransGene, Beijing, China). Subsequently, qRT-PCR was performed on the StepOnePlus™ Real-Time PCR System (Applied Biosystems, Switzerland) using *TransStart*® Top Green qPCR SuperMix (AQ132-21, TransGene). The sequences were purchased from Tsingke (Beijing, China) and are listed as follows: IMP4 (sense):5′CGAGTACCTGTACCGCAAGG -3′; (antisense),5′GGTCACACCTTCACCTCCAG -3′; E2F4: (sense):5′-GTCACAGAGGACGTGCAGAA-3′, (antisense): 5′-GCCAAGAGGGTATCTCCAGC-3′; GAPDH (sense):5′- CTCTGCTCCTCCTGTTCGAC-3′, (antisense):5′- TTCCCGTTCTCAGCCTTGAC -3′.

### 2.9. Western-Blotting

Ice-cold RIPA buffer (Cell Signaling, USA) was used to lyse the cells. Total protein lysates (50 *μ*g) were separated by denaturing 10% SDS-PAGE and electrotransfered onto a nitrocellulose membrane (Millipore, USA) for subsequent blotting with primary antibodies. The primary antibodies were: IMP4 (1 : 1000, ab181046; Abcam), Bcl-2 (1 : 1000, #15071; CST), Bax (1 : 1000, ab32503; Abcam), PARP (1 : 1000, ab191217; Abcam), E-cadherin (1 : 1000, 20874-1-AP; Proteintech, Wuhan), N-cadherin (1 : 1000, ab280375; Abcam), Vimentin (1 : 1000, ab20346; Abcam), glucose transporter 1 (GLUT1; 1 : 1000, ab115730; Abcam), hexokinase II (HK2; 1 : 1000, ab209847; Abcam), cleaved fructose phosphate kinase P (PFKP; 1 : 1000, ab32561, Abcam), pyruvate kinase M2 (PKM2; 1 : 1000, ab85555; Abcam), lactate dehydrogenase A (LDHA; 1 : 1000, #3582; Cell Signaling, USA), MEK1 (1 : 1000, ab32091; Abcam), p-MEK1 (1 : 1000, ab96379; Abcam), ERK (1 : 1000, ab184699; Abcam), p-ERK (1 : 1000, ab201015; Abcam), p21 (1 : 1000, ab109520; Abcam), p53 (1 : 1000, ab75754; Abcam), cyclin D1 (1 : 1000, ab16663; Abcam), and GAPDH (1 : 1000, ab181603; Abcam), according to the manufacturers' protocols. After incubation with secondary antibodies for 60 min, protein bands were visualised using ECL kit (Beyotime).

### 2.10. In Vivo Xenograft Model

BALB/*c* nude mice (female, 6 weeks old) were supplied by GemPharmatech (Jiangsu, China). A549 cells (3 × 10^6^) transfected with sh-NC or sh-IMP4 (Genechem) were injected subcutaneously into the left dorsal flanks. Tumour volumes were recorded every three days from the 10th day. The mice were then killed on day 30 after inoculation and the subcutaneous tumour weight of each mouse was determined.

### 2.11. H&E Staining and Immunohistochemistry

Xenograft tumours and human lung tissues were fixed in formalin, embedded in paraffin, and sectioned into 5 *μ*m thickness after embedding. For H&E staining, the sections were double stained with haematoxylin and eosin, and histopathological changes were analysed under a light microscope.

For immunohistochemistry, the sections were blocked with 5% goat serum (ZLI-9021, ZSGB-BIO, Beijing, China) for 60 min and then incubated with the primary antibodies against IMP4 (ab181046; Abcam), ki67 (ab16667; Abcam), p-MEK1 (ab96379; Abcam), and p-ERK (ab201015; Abcam) at 4°C overnight. After incubation with HRP-conjugated secondary antibody for 60 min, the sections were stained with 3′, 3-diaminobenzidine (DAB; ZLI-9017, ZSGB-BIO). Finally, the slides were observed and photographed under a light microscope.

### 2.12. Terminal Dexynucleotidyl Transferase-Mediated dUTP Nick End Labelling (TUNEL) Assay

A colorimetric TUNEL apoptosis assay kit (C1086, Beyotime) was used to assess apoptotic cells in mouse lung tissues. The lung sections were immersed in TUNEL reaction mixture in the dark at 37°C in a humidified atmosphere for 1 h. After 3 times of PBS washing, apoptotic cells in lung tissues were observed under a fluorescence microscope.

### 2.13. Dual-Luciferase Reporter Assay

The sequence of wild type or mutant IMP4 promoter including E2F4 binding sites was subcloned into a pGL3-luciferase reporter vector. The promoter sequence was synthesised by Genscript Biotech Corporation. The luciferase reporter vector and E2F4 were cotransfected into HEK-293T cells using Lipofectamine 3000 reagent (Invitrogen, USA). After culturing at 37°C for 48 hours, the cells were evaluated by a dual-luciferase reporter assay system (D0010, Solarbio, Beijing, China) following the manufacturer's instructions.

### 2.14. Bioinformatics Analysis

The Gene Expression Profiling Interactive Analysis (GEPIA) database was used to analyse patient survival data. The median was the criterion for patients to be divided into two groups on the prognosis curve. The differential expression of IMP4 across the cancer genome Atlas (TCGA) and in LUAD was performed using UALCAN according to the database obtained from TCGA. Following transfection with sh-IMP4, the transcriptome of A549 cells was sequenced by Novogene (Beijing, China). Gene set enrichment analysis (GSEA) was used to investigate the potential mechanisms by which IMP4 regulates LUAD.

Animal TFDB (http://bioinfo.life.hust.edu.cn/AnimalTFDB#!/), hTFtarget (http://bioinfo.life.hust.edu.cn/hTFtarget#!/), and Chipbase (https://rna.sysu.edu.cn/chipbase/index.php) were used to identify the transcription factors upstream of IMP4. Genes positively associated with IMP4 expression in TCGA-LUAD were analysed using cBioPortal (http://www.cbioportal.org/).

### 2.15. Statistical Analysis

The data are presented as the mean ± standard deviation (SD). Statistical analyses were performed using SPSS version 22.0. Results were compared using Student's *t*-test or one-way ANOVA. Statistical significance was set at *p* < 0.05.

## 3. Results

### 3.1. High IMP4 Expression Was Found in LUAD and Predicted Poor Prognosis for LUAD Patients

**Figure 1:**
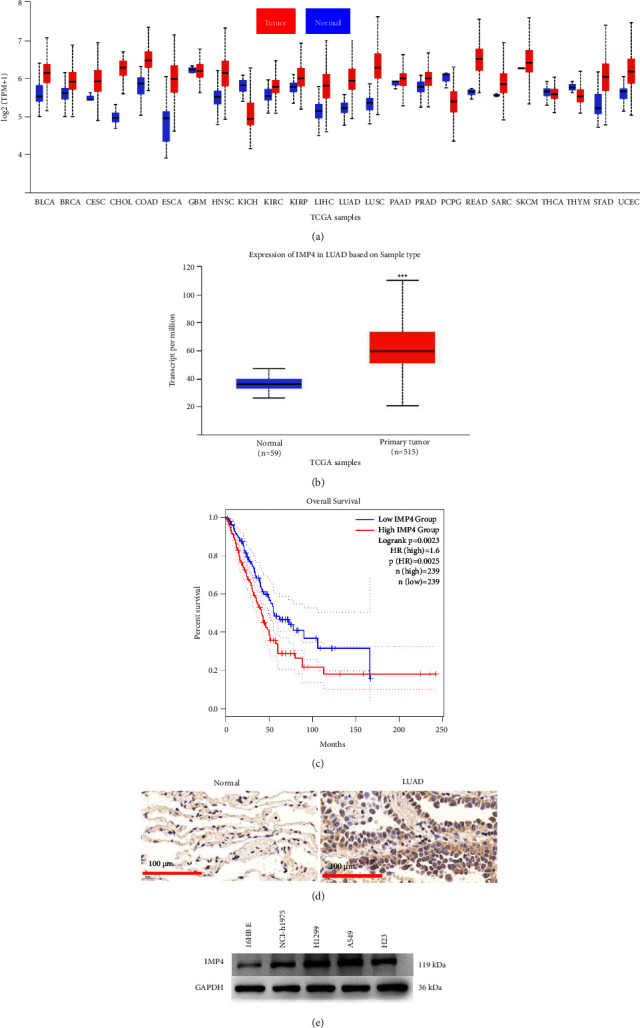
High IMP4 expression was found in LUAD and suggested association with poor prognosis for LUAD patients. (a) The differential levels of IMP4 across TCGA cancers. (b) The expression of IMP4 in LUAD was analysed using UALCAN based on TCGA database. (c) The survival curves of LUAD patients from GEPIA database. (d) Immunohistochemistry was applied to assess IMP4 levels in LUAD tissues. (d) The expression of IMP4 in 16HBE, NCI–H1975, H1299, A549, and H23 cells was determined using Western blotting.

The data from TCGA showed that IMP4 levels were up-regulated in LUAD tissues (Figures 1(a) and 1(b)) and confirmed that high IMP4 expression predicted poor prognosis in LUAD patients (Figure 1(c)). The immunohistochemistry results further demonstrated that IMP4 levels were up-regulated in LUAD patient tissues (Figure 1(d)). Additionally, we showed that the levels of IMP4 were significantly up-regulated in A549 and H1299 cells (Figure 1(e)).

### 3.2. IMP4 Silencing Inhibits Proliferation, Promotes Apoptosis, and Induces Cell Cycle Arrest in LUAD Cells

**Figure 2:**
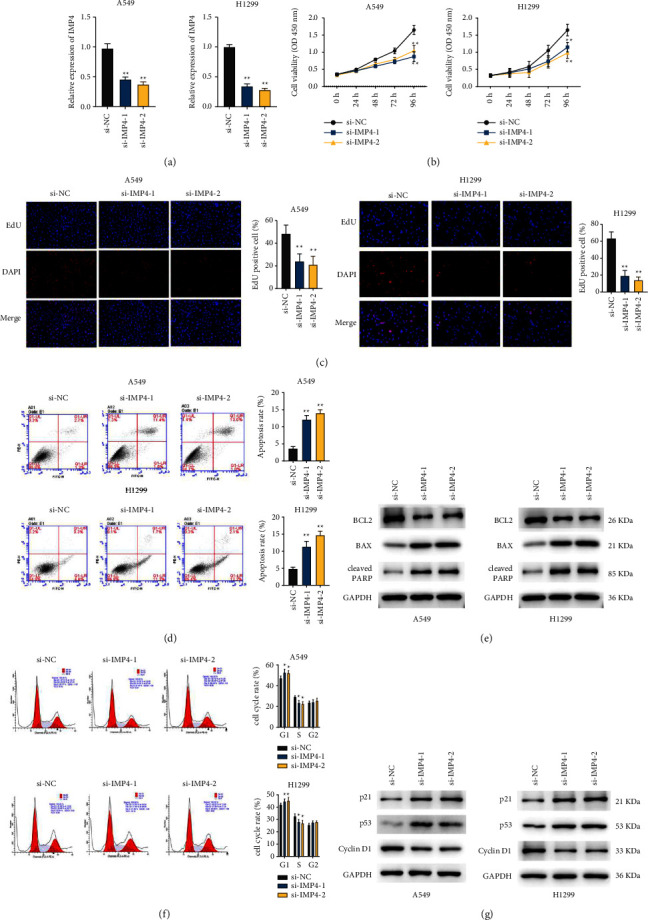
IMP4 silencing inhibited proliferation, promoted apoptosis, and induced cell cycle arrest in LUAD cells. After treatment of A549 and H1299 cells with si-IMP4-1 and si-IMP4-2, IMP4 levels were determined with qRT-PCR (a); proliferation was examined applying CKK-8 (b) and EdU analysis (c); apoptosis was detected using Annexin-V/PI double staining kit (d); the expression of Bcl-2, Bax, and PARP was measured using Western-blotting (e); cell cycle was evaluated applying cell cycle and apoptosis analysis kit (f). Protein levels of cyclin D1, P21, and P53 in A549 and H1299 cells (g). ^*∗*^*P* <  0.05, ^*∗∗*^*P*  <  0.01.

The transfection efficiency of si-IMP4-1 and si-IMP4-2 in A549 and H1299 cells was determined using qRT-PCR (Figure 2(a)). The results showed in Figures 2(b) and 2(c) revealed that IMP4 silencing notably repressed A549 and H1299 cell proliferation. Moreover, IMP4 silencing dramatically increased A549 and H1299 cell apoptosis (Figure 2(d)). To confirm its accelerating effect on apoptosis, we measured the levels of apoptosis-related proteins using Western blotting and demonstrated that the protein levels of cleaved PARP and Bax were increased, whereas Bcl-2 was decreased (Figure 2(e)). As shown in Figure 2(f), IMP4 silencing caused accumulation of A549 and H1299 cells in the G1 phase of the cell cycle. Accordingly, typical cell cycle markers such as cyclin D1 were down-regulated, whereas p21 and p53 were up-regulated upon IMP4 downregulation (Figure 2(g)). Together, these results show that IMP4 silencing represses proliferation, accelerates apoptosis, and induces cell cycle arrest in LUAD cells.

### 3.3. IMP4 Silencing Inhibits Migration and Invasion in LUAD Cells

**Figure 3:**
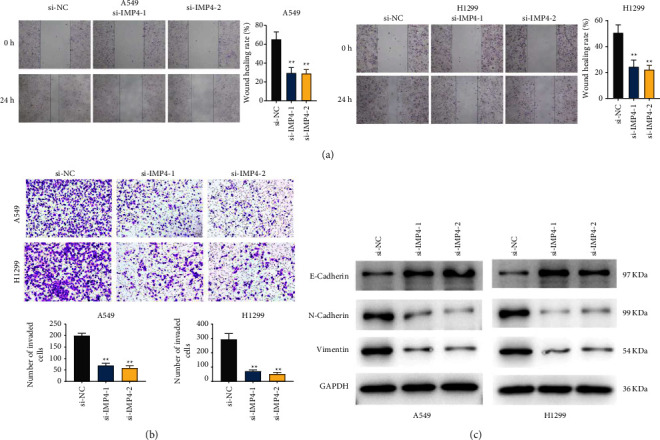
IMP4 silencing inhibited migration and invasion of LUAD cells. After transfection of A549 and H1299 cells with si-IMP4-1 and si-IMP4-2, the migration and invasion were examined by wound healing (a) and transwell assays (b); EMT-related protein expression was analysed using Western blotting (c). ^*∗∗*^*P*  <  0.01.

Data in Figures 3(a) and 3(b) show that IMP4 silencing represses the migration and invasion of both A549 and H1299 cells. To further verify this effect, we performed Western blotting and found that IMP4 silencing significantly increased E-cadherin expression and decreased N-cadherin and vimentin expression in A549 and H1299 cells (Figure 3(c)).

### 3.4. Silencing of IMP4 Inhibits Glycolysis in LUAD Cells

**Figure 4:**
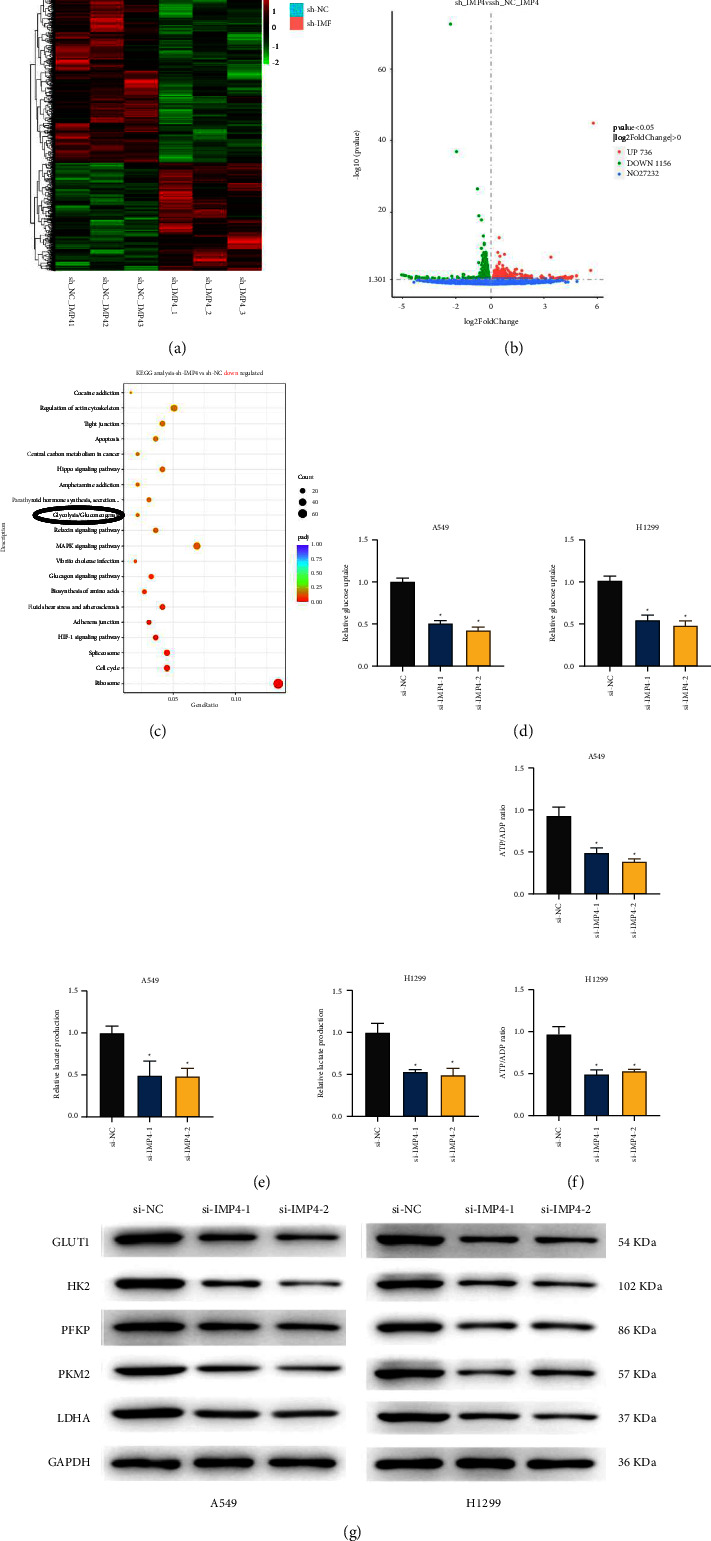
IMP4 knockdown inhibited glycolysis in LUAD cells. ((a) and (b)) The transcriptome of A549 cells transfected sh-IMP4 was sequenced by Novogene. (c) The potential pathways which IMP4 could regulate in LUAD were analysed using GSEA. After treatment of A549 and H1299 cells with si-IMP4-1 and si-IMP4-2, glucose uptake (d), lactate production (e), and ATP generation (f) were evaluated; the expression of GLUT1, HK2, PFKP, PKM2, and LDHA was analysed by Western blotting (g). ^*∗*^*P*  <  0.05.

Following transfection with sh-IMP4, the transcriptome of A549 cells was sequenced by Novogene (Beijing, China) (Figures 4(a) and 4(b)). GSEA analysis revealed that several processes were down-regulated by sh-IMP4, including glycolysis (Figure 4(c)). As shown in Figures 4(d)–4(f), silencing IMP4 significantly decreased glucose uptake, lactate secretion, and ATP production. Moreover, the expressions of glycolysis-related genes, including GLUT1, HK2, PFKP, PKM2, and LDHA, were markedly reduced by IMP4 silencing (Figure 4(g)). These results show that IMP4 silencing can inhibit glycolysis in LUAD cells.

### 3.5. Silencing of IMP4 Inhibits Tumour Growth and Glycolysis in a Nude Mouse Xenograft Model

**Figure 5:**
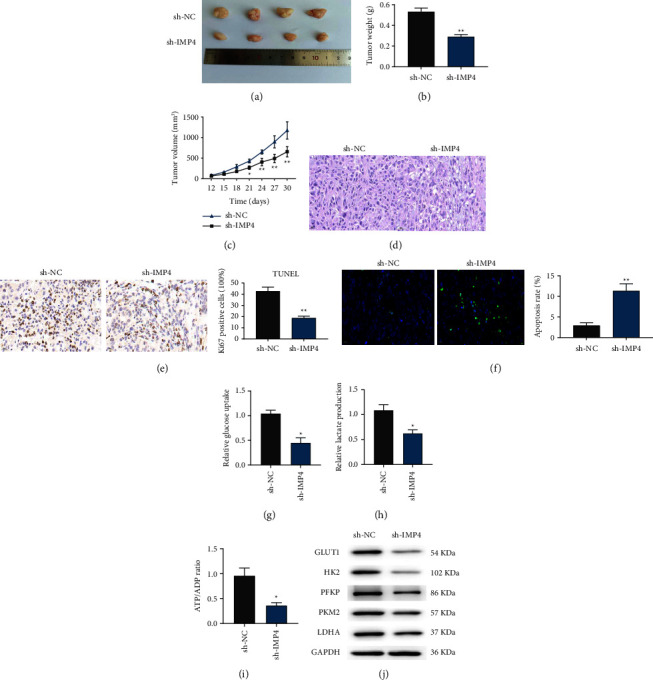
IMP4 knockdown inhibited tumour growth in a nude mouse xenograft model. (a) Photographs of subcutaneous tumours following different treatments. The tumour weight (b) and volume (c) of subcutaneous tumours. (d) The histopathological changes in subcutaneous tumour tissues were analysed with H&E staining. (e) Immunohistochemistry was applied to assess Ki67 levels in subcutaneous tumours. (f) TUNEL assay was used to evaluate apoptosis-related TUNEL index in subcutaneous tumour tissues. Glucose uptake (g), lactate production (h), and ATP generation (i) were evaluated in subcutaneous tumours; expression of GLUT1, HK2, PFKP, PKM2, and LDHA was analysed using Western blotting (j)^*∗*^*P*  <  0.05, ^*∗∗*^*P*  <  0.01.

As shown in Figures 5(a)–5(c), IMP4 silencing notably reduced the volume and weight of subcutaneous tumours in the A549-derived tumour mouse model. Moreover, H&E staining confirmed that IMP4 silencing decreased the number of LUAD cells in tumours (Figure 5(d)). Immunohistochemistry results showed that Ki67 expression in the sh-IMP4 group was significantly lower than that in the sh-NC group (Figure 5(e)). In addition, TUNEL results revealed that IMP4 silencing significantly promoted the apoptosis-related TUNEL index (Figure 5(f)). Furthermore, IMP4 silencing significantly decreased glucose uptake, lactate secretion, and ATP production *in vivo* (Figures 5(g)–5(i)). Moreover, the expression of glycolysis-related genes, including GLUT1, HK2, PFKP, PKM2, and LDHA was markedly reduced by IMP4 silencing (Figure 5(j)). These results suggest that IMP4 silencing inhibits tumour growth and glycolysis in a nude mouse xenograft model.

### 3.6. Silencing of IMP4 Suppresses the Malignant Phenotypes Via Inhibiting the ERK Pathway

**Figure 6:**
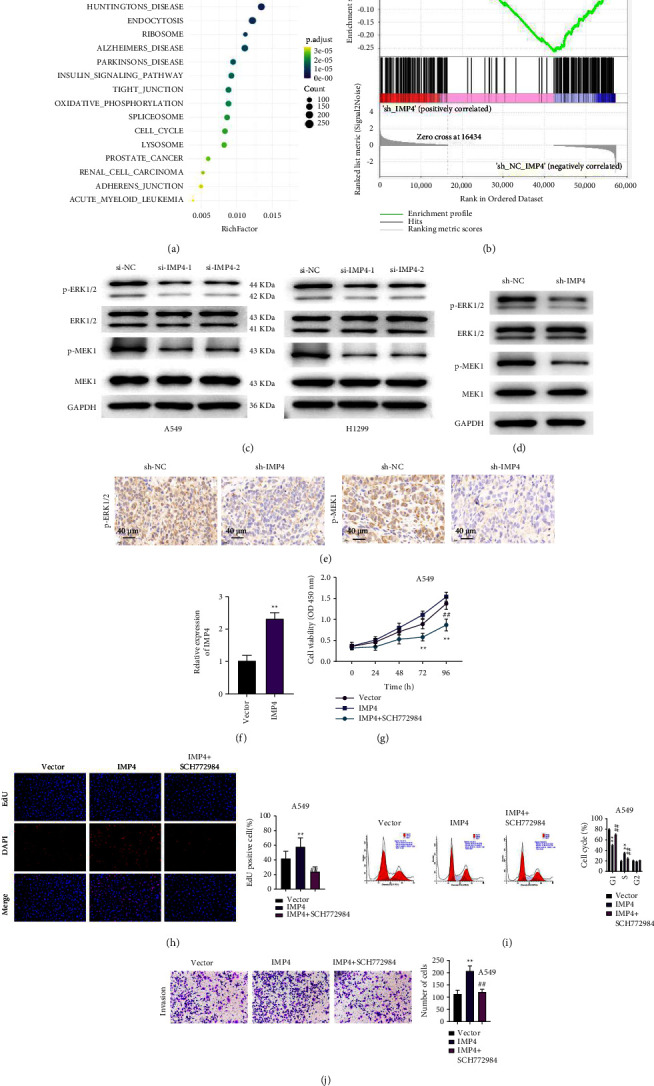
IMP4 overexpression promoted the malignant phenotypes via activating the ERK pathway in LUAD cells. ((a) and (b)) The potential pathways which IMP4 could regulate in LUAD were analysed using GSEA. (c) After transfection of A549 and H1299 cells with si-IMP4-1 and si-IMP4-2, the expression of MEK, p-MEK, ERK, and p-ERK was analysed using Western blotting. (d) The expression of MEK, p-MEK, ERK, and p-ERK in subcutaneous tumour tissues were analysed using Western blotting. (e) Immunohistochemistry was applied to assess p-ERK1/2 and p-MEK1 levels in subcutaneous tumour. (f) After transfection of A549 cells with pcDNA3.1-IMP4, qRT-PCR was applied to assess IMP4 levels. After transfection with pcDNA3.1-IMP4 or treatment with SCH772984, in A549 cell proliferation was evaluated using the CKK-8 (g) and EdU (h); (i) After transfection with pcDNA3.1-IMP4 or treatment with SCH772984, A549 cell cycle was assessed using flow cytometry. (j) After transfection with pcDNA3.1-IMP4 or treatment with SCH772984, A549 cell invasion was assessed using transwell. ^*∗*^*P*  <  0.05, ^*∗∗*^*P*  <  0.01; ^#^*P*  <  0.05, ^##^*P*  <  0.01.

GSEA analysis revealed that several signalling pathways were down-regulated by sh-IMP4, including the MAPK pathway (Figures 6(a) and 6(b)). We selected the ERK pathway, one of the MAPK pathways, for further investigation. As shown in Figure 6(c), IMP4 silencing decreased the expression of p-MEK and p-ERK in A549 and H1299 cells (Figure 6(c)). We analysed the transfection efficiency of pcDNA3.1-IMP4 in A549 cells (Figure 6(f)). Moreover, IMP4 overexpression dramatically elevated proliferation and invasion, and induced accumulation of A549 cells in the S phase of the cell cycle (Figures 6(g)–6(j)). Meanwhile, knockdown of IMP4 significantly reduced p-MEK and p-ERK expression in xenograft tumours (Figures 6(d) and 6(e)). All these functions induced by IMP4 overexpression were reversed following the treatment of A549 cells with SCH772984 (an inhibitor of the ERK pathway) (Figures 6(e)–6(h)).

### 3.7. E2F4 Up-Regulates IMP4 and Promotes the Progression of LUAD

**Figure 7:**
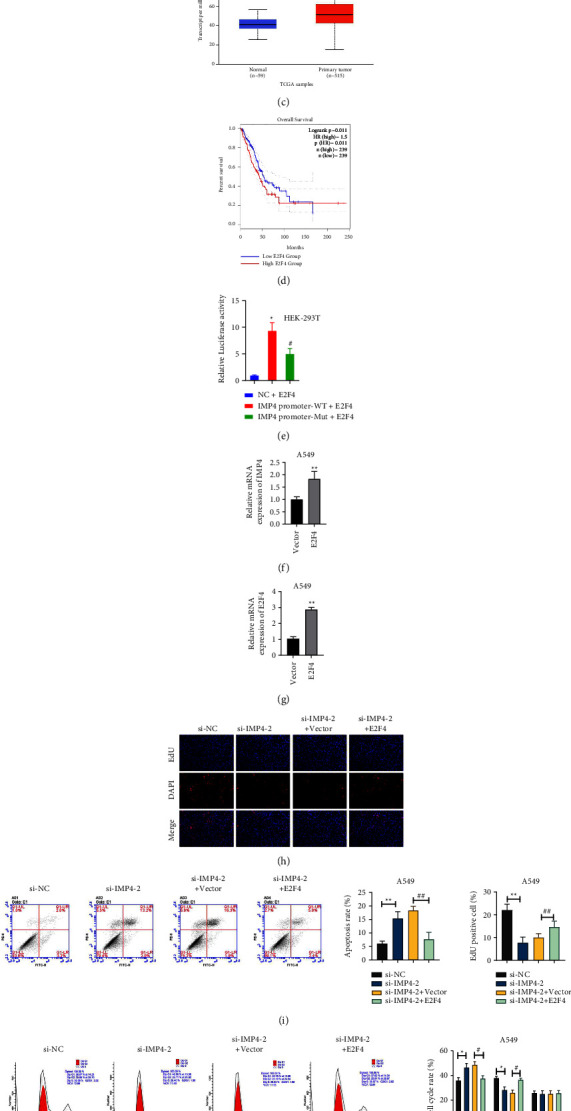
E2F4 up-regulated IMP4 and promoted the progression of LUAD. (a) Venn chart identified E2F4 via analysing the Animal TFDB, hTFtarget, and Chipbase among the genes positively associated with IMP4 expression in TCGA-LUAD. (b) TCGA-LUAD database verified that IMP4 was positively related to E2F4 expression. (c) The expression of E2F4 in LUAD was analysed using the UALCAN based on TCGA database. (d) The survival curves of LUAD patients from GEPIA database. (e) Luciferase activities were evaluated via performing dual luciferase reporter assay. After pcDNA3.1-E2F4 vector was transfected into A549 cells, the mRNA levels of IMP4 (f) and E2F4 (g) were determined using qRT-PCR. After pcDNA3.1-E2F4 vector and si-IMP4-2 co-transfection, A549 cell proliferation was analysed using the EdU assay (h); apoptosis was assessed using FITC-Annexin V/PI apoptosis detection kit (i); the cell cycle was evaluated using cell cycle and apoptosis analysis kit (j); invasion was examined using the transwell assay (k). ^*∗*^*P*  <  0.05, ^*∗∗*^*P*  <  0.01, ^##^*P*  <  0.01.

The Venn chart identified E2F4 in the Animal TFDB, hTFtarget, Chipbase, and the genes positively associated with IMP4 expression in TCGA-LUAD (Figure 7(a)). The TCGA-LUAD database showed that IMP4 positively correlated with E2F4 levels (Figure 7(b)). Moreover, the data from TCGA demonstrated that E2F4 levels were up-regulated in LUAD tissues (Figure 7(c)) and further confirmed that high E2F4 expression predicted poor prognosis in LUAD patients (Figure 7(d)). To further verify that E2F4 mediates upregulation of IMP4, a dual luciferase reporter assay was performed. As seen in Figure 7(e), luciferase activity was increased in the IMP4 promoter-WT + *E*2F4 group compared to the NC + *E*2F4 group. Meanwhile, luciferase activity was decreased in the IMP4 promoter-MUT + *E*2F4 group compared with the IMP4 promoter-WT + *E*2F4 group. Moreover, after A549 cells were transfected with the pcDNA3.1-E2F4 vector, IMP4 expression was significantly elevated (Figure 7(f)), further indicating that IMP4 is positively correlated with E2F4 expression in LUAD cells. Subsequently, the transfection efficiency of the pcDNA3.1-E2F4 vector in A549 cells was analysed using qRT-PCR (Figure 7(g)). Furthermore, the results presented in Figures 7(h)–7(k) show that the up-regulated E2F4 significantly reversed the inhibitory effect of IMP4 knockdown on the malignant phenotypes of A549 cells. These results show that E2F4 upregulates IMP4 and promotes the progression of LUAD.

## 4. Discussion

**Figure 8:**
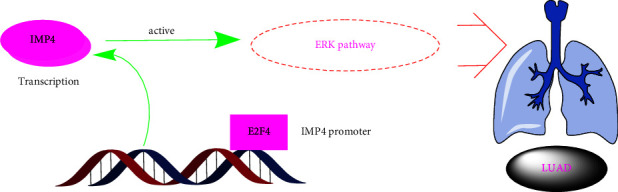
IMP4 promotes lung adenocarcinoma progression through activating the ERK pathway.

Owing to the adverse results reported in cancer statistics, there is an urgent need to identify new markers for LUAD [2]. A previous study showed that IMP4 is highly expressed in human lung cancer and verified it as a new therapeutic target for lung cancer [5]. In this study, we hypothesised that IMP4 can promote the progress of LUAD by activating the ERK pathway (Figure 8). The experimental results confirm our hypothesis that IMP4 was overexpressed in LUAD, meanwhile the silencing of IMP4 significantly inhibited proliferation, migration, invasion, and glycolysis; promoted apoptosis and induced LUAD cell cycle arrest; and suppressed tumour growth and glycolysis in a nude mouse xenograft model. Subsequently, we investigated the IMP4-related molecular mechanisms in LUAD cells.

Tumour cells can undergo unrestricted divisions and proliferation. Rapid proliferation of cancer cells requires rapid energy expenditure. Glycolysis in tumour cells accelerates glucose uptake and lactic acid generation, resulting in an acidic environment that induces rapid proliferation and metastasis [12–15]. Moreover, glycolysis is reported to be involved in ATP synthesis and cell pathway activation [16]. Some genes have been reported to be related to glycolysis in cancers [17, 18]. For instance, PPFIA4 has been shown to promote colon cancer cell proliferation and migration by enhancing tumour glycolysis [19]. PFK1 has been shown to accelerate proliferation and migration, and reduce radiosensitivity by promoting glycolysis in colorectal cancer [20]. In this study, we demonstrated that IMP4 dramatically increased glucose uptake, lactate generation, and ATP synthesis in LUAD cells and tumour tissues, suggesting that IMP4 could enhance glycolysis in LUAD. To further verify this hypothesis, the expression levels of glucose transporters and key glycolytic enzymes, including GLUT1, HK2, PFKP, PKM2, and LDHA, were detected by Western blotting. The results revealed that IMP4 silencing reduced the levels of GLUT1, HK2, PFKP, PKM2, and LDHA in LUAD cells and tumour tissues.

In recent years, increasing number of studies have shown that the activation of ERK signalling can contribute to the growth, invasion, and epithelial-mesenchymal transition in tumour cells [21, 22]. In addition, a variety of genes can play important roles in LUAD by regulating ERK signalling. For example, DLC1 inhibits LUAD cell proliferation and invasion by repressing MAPK/ERK signalling [23]. Another study confirmed that FANCI can act as an oncogene in LUAD by cooperating with IMPDH2 to accelerate cell proliferation through activation of the MEK/ERK pathway [24]. ERK signalling has recently emerged as a critical modulator of glycolysis during tumorigenesis [9]. He et al. [25] reported that PGK1 contributes to the growth of renal clear cell carcinoma by activating CXCR4/ERK signalling and promoting glycolysis. In this study, we demonstrated that IMP4 silencing inhibited the activation of ERK signalling in LUAD cells. In addition, to further verify this hypothesis, we performed rescue experiments using IMP4 overexpression and the ERK pathway inhibitor SCH772984. We found that the effects of IMP4 overexpression were reversed following treatment with SCH772984.

## 5. Conclusion

In our study, we demonstrated that IMP4 silencing significantly inhibited proliferation, migration, invasion, and glycolysis; promoted apoptosis; induced cell cycle arrest in LUAD cells; and suppressed tumour growth and glycolysis in a nude mouse xenograft model. We also showed that IMP4 silencing suppressed the malignancy of LUAD by inactivating ERK signalling. Our study provides strong evidence that IMP4 is an outstanding diagnostic marker as well as a potential therapeutic target for LUAD.

## Data Availability

The datasets used and/or analysed during the current study are available from the corresponding author on reasonable request.
